# Properties of EPDM Nanocomposites Reinforced with Modified Montmorillonite

**DOI:** 10.3390/polym16162381

**Published:** 2024-08-22

**Authors:** Zhanxu Li, Zilong Chen, Weichong Sun, Yangling Liu, Xiong Wang, Jun Lin, Jian Wang, Shaojian He

**Affiliations:** 1 State Key Laboratory of Alternate Electrical Power System with Renewable Energy Sources, North China Electric Power University, Beijing 102206, China; zhanxuli0614@163.com (Z.L.); zilongchen86@163.com (Z.C.); 13811157813@163.com (W.S.); liuyangling010223@163.com (Y.L.); jun.lin@ncepu.edu.cn (J.L.); wangjian31791@ncepu.edu.cn (J.W.); 2 South China Institute of Environmental Sciences, Ministry of Ecology and Environment, Guangzhou 510655, China

**Keywords:** ethylene propylene diene monomer, montmorillonite, polydopamine, vinyltrimethoxysilane, gel compounding method

## Abstract

Considering the dilemma of obtaining economic and high-performance composites based on non-polar and main-chain-saturated ethylene propylene diene monomer rubber (EPDM), we proposed an effective and universal filler modification and nanocomposite preparation method. Specifically, the montmorillonite (MMT) surface was coated with polydopamine (PDA) to obtain DMMT, which was confirmed by XRD, XPS, FTIR, and TGA. After compounding DMMT gel with the solid EPDM via the gel compounding method, a silane coupling agent, vinyltrimethoxysilane, was introduced to construct covalent interactions between rubber and filler. Compared with the unmodified MMT filler EPDM, the EPDM/DMMT nanocomposite showed much fewer filler aggregates in the matrix. The highest tensile strength of the composites reached 6.5 MPa with 10 phr DMMT, almost 200% higher than that of pure EPDM.

## 1. Introduction

Ethylene propylene diene monomer rubber (EPDM) is widely used in automobiles, seals, and other applications due to its excellent heat and weather resistance [1,2,3]. Since vulcanized EPDM made without reinforcement agents has a low mechanical strength, it cannot be applied in the engineering field unless effective reinforcing treatment is carried out [4]. Nonetheless, it is difficult to construct a strong interface interaction in non-polar EPDM composites by directly incorporating reinforcing agents. The lack of polar functional groups makes it difficult to uniformly disperse reinforcing agents in the EPDM matrix, resulting in an unsatisfactory reinforcement effect. Consequently, the organic modification of filler is a common strategy to overcome these deficiencies. For example, coupling agents are often used to modify silica [5], and grafting strategies are often employed to modify carbon black [6]. Nevertheless, those fillers are relatively expensive, and at the same time, the modification processes are often complex, which limits their application in engineering.

As a result of its low price, high aspect ratio, and good mechanical properties, montmorillonite (MMT) is expected to replace traditional reinforcing agents, such as carbon black and silica, in elastomers [7,8,9,10]. To obtain high-performance polymer/MMT nanocomposites, good dispersion of the MMT layer and suitable polymer–MMT interfacial interactions are essential [11,12,13,14]. MMT, however, is easily aggregated in polymer matrixes due to the poor compatibility between hydrophilic montmorillonite and hydrophobic matrixes [15,16,17]. In particular, for EPDM/MMT composites, it is more difficult to achieve the uniform dispersion of MMT in EPDM due to the lack of polar groups [18,19]. Because the EPDM main chain is highly saturated, it is difficult to design the interface between EPDM and montmorillonite, especially the covalent interface. In the above cases, the method of modifying the filler with a compatibilizer is generally used to change the surface adhesion. For example, multifunctional modifiers [20] and grafted polymer brushes [21] can be used. 

Polydopamine (PDA), a substance that forms from dopamine in aqueous solutions by self-polymerization, adheres closely to various substrates and is widely used to modify surfaces [22,23,24,25]. Furthermore, hydroxyl groups used in PDA coating offer the potential to introduce multiple functional groups through reactions, allowing further surface modification [26,27]. PDA-coated MMT (DMMT) layers are easily exfoliated in water, and so directly blending rubber latex with a DMMT suspension to prepare composites is a feasible way to reduce DMMT aggregation and functional group embedding [28,29]. It is of practical significance to find a method with which to prepare EPDM/DMMT composites with good dispersion of DMMT; since EPDM lacks a latex form, this will also provide a new path for the preparation of solid rubber/lamellate filler composites.

In previous studies [30,31,32], we developed an effective method, called the gel compounding method, that can obtain good filler dispersion and high-performance nanocomposites. Herein, we proposed an effective and universal method with which to prepare high-performance EPDM/MMT nanocomposites using DMMT. An EPDM/DMMT nanocomposite was obtained by the gel compounding method. Specifically, the general experimental process is as follows: EPDM was compounded with vinyltrimethoxysilane (VTMS) and DMMT gel successively using a two-roll mill. The mixture was vacuum-dried and then we followed the conventional rubber processing procedure to prepare EPDM/DMMT nanocomposite. Finally, an EPDM/DMMT nanocomposite with superior DMMT dispersion and high interface strength was obtained. This provides a feasible path for the preparation of high-performance non-polar rubber/sheet filler composites.

## 2. Experimental Section

### 2.1. Materials

Sodium montmorillonite (MMT) with a cation exchange capacity of 93 mequiv/100 g was supplied by Liufangzi Clay Factory, Jilin, China. EPDM 4045 (ENB type) was supplied by Jilin Chemical Industry Co., Ltd., Jilin, China. Dopamine hydrochloride (Dopa, 98%) was purchased from Innochem and tris(hydroxymethyl)aminomethane (Tris, 99.8%) was purchased from Alfar Aser. Other compounding ingredients obtained, including vinyltrimethoxysilane (VTMS), zinc oxide (ZnO), magnesium oxide (MgO), N-isopropyl-N′-phenyl-p-phenylenediamine (4010NA), triallyl isocyanurate (TAIC), and dicumyl peroxide (DCP), were commercial-grade products. All materials were used as received. 

### 2.2. Preparation of DMMT Gel

Purified MMT was obtained according to the procedure outlined in our previous work [33]. Then, a given amount of purified MMT was stirred in deionized water under vigorous stirring conditions for 2 h to obtain a suspension with a concentration of 2 wt%. Dopamine hydrochloride (1.5 g/L) and Tris (1.2 g/L, with the pH adjusted to 8.5) were added into the suspension, followed by continuous agitation for 4 h at ambient temperature. The product was centrifuged at 10,000 rpm for 15 min and subsequently washed with deionized water at least five times. After centrifugation, a product named DMMT gel with a concentration of about 10 wt% was obtained, which was directly blended with EPDM. Purified MMT was swelled in deionized water at a concentration of 10 wt% by vigorous stirring for 2 h to obtain DMMT gel. 

### 2.3. Preparation of EPDM/DMMT Nanocomposites

**Scheme 1:**
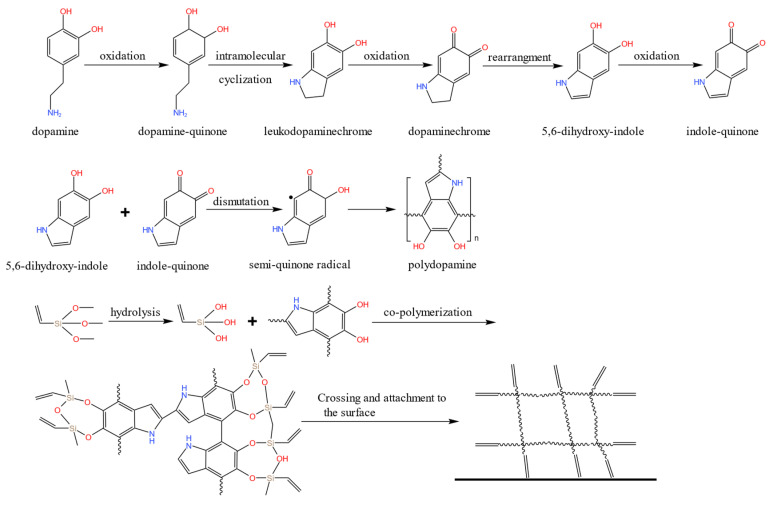
Schematic illustration of mechanism for polymerization of dopamine and interfacial co-crosslinking mechanism of EPDM/DMMT-10.

Then, a given amount of DMMT gel was mixed with EPDM in a two-roll mill to obtain an EPDM/DMMT gel composite. To remove the water, the gel compound was rolled into a thin sheet and subsequently vacuum-dried at 80 °C for 48 h to obtain an EPDM/DMMT nanocomposite. Then, an EPDM/DMMT nanocomposite was mixed with the curing agents. Finally, an EPDM/DMMT nanocomposite was vulcanized at 160 °C with optimal vulcanization time to obtain an EPDM/DMMT nanocomposite. For comparison, EPDM/MMT nanocomposite was prepared by replacing DMMT gel with 10 wt% MMT gel according to the above process. The recipes (parts by weight) for EPDM composites are shown in Table 1. And the interfacial co-crosslinking mechanism of EPDM/DMMT-10 and mechanism for the polymerization of dopamine are illustrated in Scheme 1.

### 2.4. Characterization and Measurements

Fourier transform infrared spectra (FTIR) of MMT, DMMT and PDA were recorded using a Thermo Scientific Nicolet iS20 spectrometer (Thermo Fisher Scientific Inc., Waltham, MA, USA) with a resolution of 4 cm^−1^. X-ray photoelectron spectroscopy (XPS) measurements were performed on Thermo Scientific K-Alpha XPS spectrometer (Thermo Fisher Scientific Inc., Waltham, MA, USA) with an Al Kα X-ray source. All the binding energies (B.E.) obtained from the XPS analysis were corrected using the C 1s at 284.6 eV. Thermogravimetric analysis (TG) was conducted using a TGA 550 analyzer (TA Instruments, New Castle, DE, USA) under a nitrogen atmosphere from 45 to 800 °C with a heating rate of 10 °C min^−1^. X-ray diffraction (XRD) experiments involving EPDM nanocomposites were carried out on a diffractometer (D/Max-III C, Rigaku, Tokyo, Japan) with Cu Kα radiation. The morphological structure of the EPDM composites’ tensile-fractured surfaces was observed using scanning electron microscopy (SEM) (FEI Quanta200F, Hitachi Co., Tokyo, Japan) at an accelerating voltage of 5 kV. The tensile properties were assessed by means of a universal test instrument (GT-TC2000, Gotech Testing Machines Inc., Taiwan, China) according to ISO 37-2011 [34]. Dynamic mechanical analysis (DMA) of EPDM composites was performed on a TA Q800 instrument (TA Co., Ltd., Newcastle, DE, USA) under a nitrogen atmosphere. The samples were assessed in tensile mode at 1 Hz and heated at a rate of 2 °C min^−1^. 

## 3. Results and Discussion

### 3.1. DMMT Characterization

**Figure 1:**
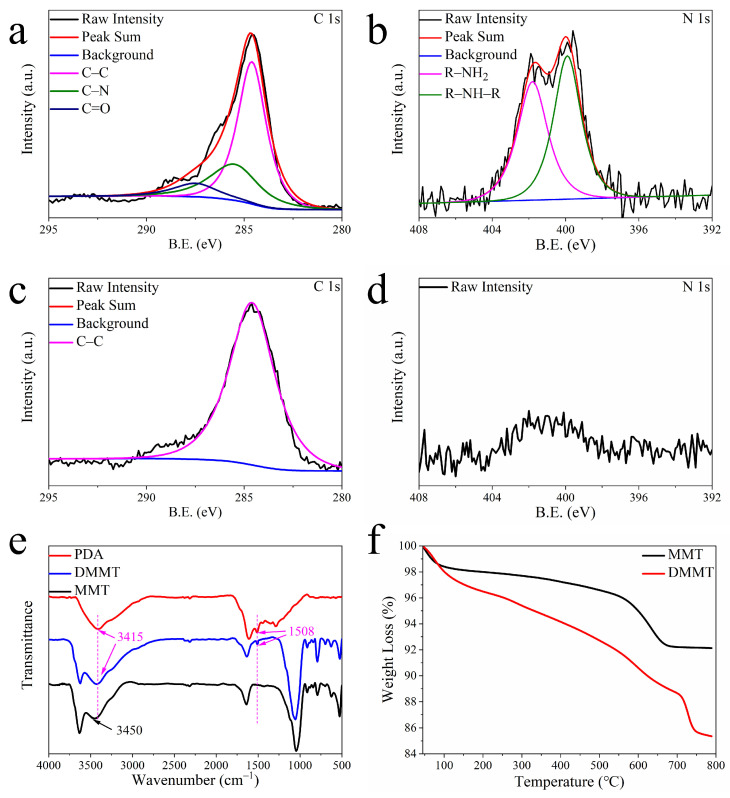
XPS spectra of (**a**,**b**) DMMT and (**c**,**d**) MMT; FTIR spectra (**e**) of PDA, DMMT and MMT; TGA curves (**f**) of MMT and DMMT.

**Figure 2:**
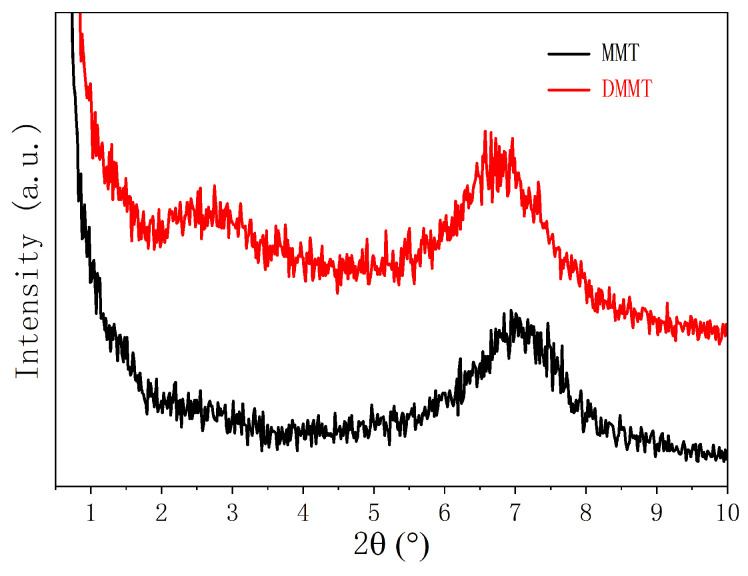
XRD curves of MMT and DMMT.

As illustrated in Figure 1a, the C 1s core-level spectrum of DMMT has three functional peaks, corresponding to C-C species at 284.6 eV, C-N species at 285.8 eV, and C=O species at 287.4 eV. For comparison, the C 1s spectrum of MMT (Figure 1c) only has one peak, corresponding to the C-C species. The N 1s core-level spectrum of DMMT (Figure 1b) can be curved-fitted with two peak components, with B.E. at 401.8 eV for the R–NH2 species and 399.9 eV for the R_1_-NH-R_2_ species [35]. However, there are no characteristic peaks on the N 1s spectrum of MMT (Figure 1d). The functional groups containing C and N indicate that MMT was successfully modified by PDA. Furthermore, FTIR was used to probe the successful modification of PDA on MMT. As shown in Figure 1e, DMMT exhibited two bands around 3415 cm^−1^ and 1508 cm^−1^, attributable to catechol O-H and N-H from PDA, respectively [36]. The characteristic bands of DMMT further confirm that PDA was coated on MMT, which is consistent with the XPS results. TGA curves (Figure 1f) show that DMMT has one more weightlessness process than MMT, which is caused by the decomposition of PDA. The content of PDA coating on the surface of MMT was calculated to be ~7.0 wt%. The XRD curves of MMT and DMMT are compared in Figure 2. Diffraction peaks appeared at both 2.6° and 7.2° in DMMT, indicating increased layer spacing, which occurs due to the existence of PDA macromolecules between DMMT layers [37]. 

### 3.2. Structure and Morphology of EPDM Composites

**Figure 3:**
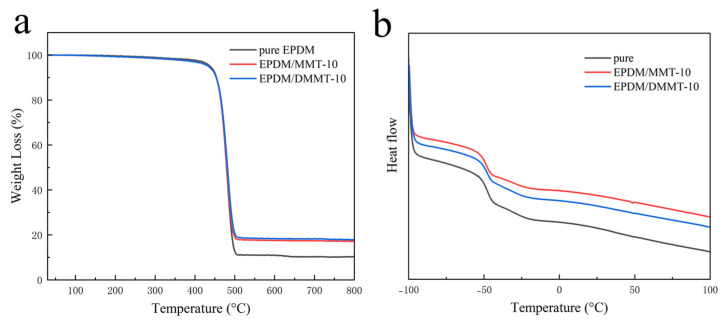
TGA curves (**a**) of pure EPDM, EPDM/MMT-10, and EPDM/DMMT-10; DSC curves (**b**) of pure EPDM, EPDM/MMT-10, and EPDM/DMMT-10.

Figure 3a shows TGA curves. In the figure, we can see that EPDM/MMT-10 and EPDM/DMMT-10 exhibited similar pyrolysis behaviors. The weight loss percentage of EPDM/MMT-10 and EPDM/DMMT-10 was lower than that of pure EPDM rubber. The results showed that MMT and DMMT were well dispersed in rubber and had improved thermal stability [38]. Figure 3b shows DSC curves. As can be seen from the curves, the glass transition temperature of EPDM/DMMT and EPDM/MMT is basically the same as that of EPDM [23].

**Figure 4:**
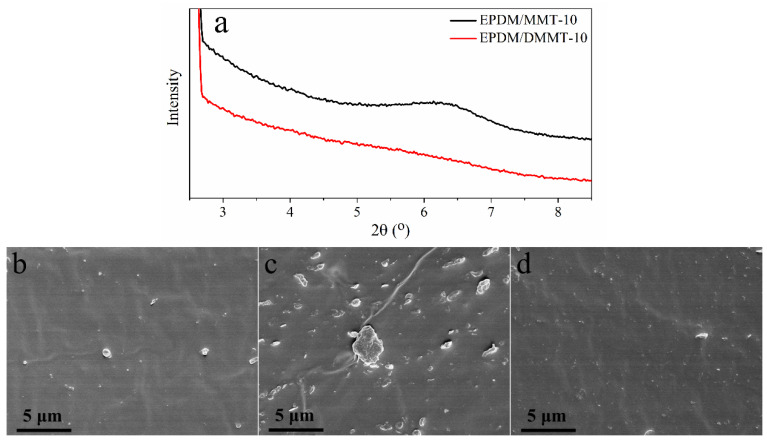
XRD curves (**a**) of EPDM/MMT-10 and EPDM/DMMT-10; SEM images of (**b**) pure EPDM, (**c**) EPDM/MMT-10, and (**d**) EPDM/DMMT-10.

In Figure 4a, EPDM/MMT-10 shows a diffraction peak at 6.2°, corresponding to a layer spacing of 1.43 nm. Compared to the pristine MMT, the slight expansion of layer spacing is similar to that seen in our previous study [35,39]. This is attributed to the incomplete removal of the water remaining in the MMT layers. When the DMMT is incorporated into EPDM, no diffraction peak is found in the XRD curve. In the stage of EPDM/DMMT-10 nanocomposite preparation, the aggregation of MMT in the hydrophobic matrix is weakened by water and PDA coating, and the compatibility between MMT and EPDM is increased by PDA coating. Even in the vulcanization process with high pressures and high temperatures, the movement of chain segments is enhanced, but the chemical bond is constructed by the PDA, VTMS, and EPDM matrixes on the MMT surface. This restricts the sliding out of macromolecules between layers, and so no diffraction peak exists in the XRD curve.

In Figure 4b, the EPDM composite has a dense and smooth surface without defects. In Figure 4c, with the addition of MMT, the size and distribution of MMT aggregates are uneven, and the clear interface of MMT–EPDM indicates that the interface interaction of MMT–EPDM is weak. As shown in Figure 4d, with the addition of DMMT, the aggregates vanish, and the DMMT–EPDM boundaries blur. This comparison shows that DMMT–EPDM has a strong interfacial interaction. The PDA coating on the MMT surface contains a large number of hydroxyl groups, which can be connected to the silane coupling agent (VTMS) by a grafting reaction, while the double bonds contained in VTMS are co-crosslinked with the EPDM matrix during the curing process, which significantly improves the interface interaction [29,40]. 

### 3.3. Properties of EPDM Composites

**Figure 5:**
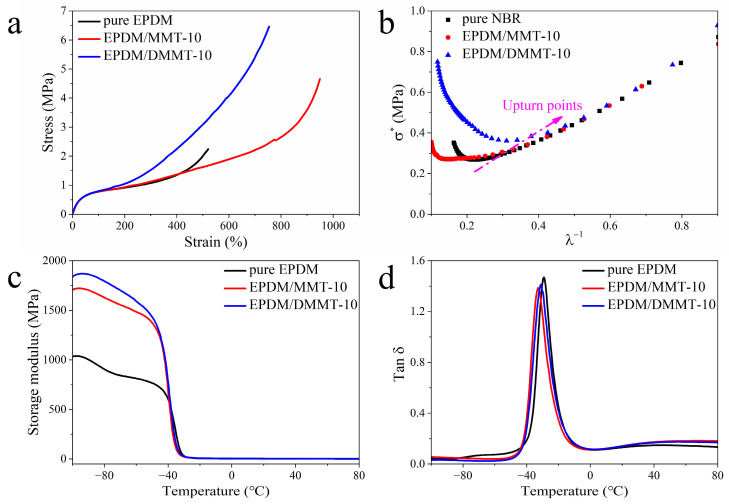
(**a**) Stress–strain curves, (**b**) reduced stress (σ*)–λ^−1^ curves, (**c**) storage modulus–temperature curves, and (**d**) tanδ–temperature curves of EPDM composites.

Figure 5a shows the stress–strain curves of EPDM composites. It can be clearly observed that the tensile strength and the elongation at break of all the nanocomposites improved. For example, the tensile strength for pure EPDM is 2.2 MPa; this value increases to 4.7 MPa and 6.5 MPa for EPDM/MMT-10 and EPDM/DMMT-10. However, EPDM/DMMT nanocomposite has a different stress–strain behavior, and the stress increases rapidly when the strain is more than 200%. This can be attributed to the fact that PDA coating on the MMT surface is linked to VTMS by chemical bonding, which successfully introduces a double bond on the MMT surface and causes a connection to the macromolecular chain during curing. In addition, the excellent dispersion of the DMMT the of EPDM matrix also contributes to the improvement of mechanical properties.

The Mooney–Rivlin equation was used to evaluate the elastomer network and the interface interaction between EPDM and DMMT as follows:

$$\sigma^{*}=\frac{\sigma }{\lambda -\lambda^{-2}}=2C_{1}+2C_{2}\lambda^{-1}$$

where *σ* is applied stress, *λ* is the extension ratio, and *C*_1_ and *C*_2_ are the Mooney–Rivlin constants. As elaborated in Figure 5b, during the process of stretching, the stress decreases first and then increases, and these upturn points are related to the anchoring of the packing to the rubber macromolecular chain. The upturn occurs at smaller deformations for EPDM/DMMT-10 than for EPDM/MMT-10. This phenomenon is ascribed to DMMT serving as the crosslinking center and forming strong chemical interactions, which leads to more rubber chains being introduced into the interface, and these rubber chains are easily oriented during the stretching process. 

To investigate the DMMT–EPDM interfacial interaction, dynamic mechanical analysis was carried out. As illustrated in Figure 5c, with the addition of filler into the EPDM matrix, the storage modulus increased regardless of whether the MMT was modified or not. However, the storage modulus of EPDM/DMMT-10 is higher than that of EPDM/MMT-10. The results demonstrated that DMMT has a stronger limiting effect on EPDM molecule under the same curing agent loading, which is attributed to the superior dispersion of DMMT on the matrix and the strong interfacial interaction between DMMT and EPDM. The addition of DMMT or MMT reduces the tanδ value of the composite, which is due to the reduction in the free volume of the rubber molecular chain after the addition of filler, thus limiting the movement of the polymer chain segment. It is worth noting that the tanδ of EPDM/DMMT-10 is 1.41, which is 0.02 higher than that of EPDM/MMT-10. The energy dissipation of PDA macromolecular chains on the MMT surface under small deformation forces and the interaction of hydrogen and covalent bonds at the interface of DMMT–EPDM limit the movement of macromolecular network segments. The combined effect of these two factors ultimately leads to subtle differences in tanδ values. However, the Tg values of EPDM/DMMT-10 and EPDM/MMT-10 nanocomposites were almost constant compared to pure EPDM, which is consistent with other reported results for non-polar rubber/MMT composites [1,41].

Table 2 shows the mechanical properties of various filler-filled EPDM composites in the literature. The reported filler EPDM composites made with carbon black or aluminum hydroxide show a tensile strength of ~7.2 MPa; however, quite a lot of filler was introduced. The EPDM composite filled with 25 phr silica shows a tensile strength of only 1.8 MPa, and the composites filled with 10 phr modified MMT exhibit a tensile strength of ~4.3 MPa. For comparison, the EPDM composite filled with 10 phr DMMT in this study shows a tensile strength of 6.5 MPa, and shows an elongation at break of 720%, which is much higher than that seen in the literature. 

## 4. Conclusions

In this study, the surface modification of MMT by PDA and vinyltrimethoxysilane grafting was successfully demonstrated. XRD, XPS, FTIR, and TGA confirmed that the montmorillonite (MMT) surface could be coated with polydopamine (PDA) to obtain DMMT. Subsequently, EPDM composites were prepared by the gel compounding method. Compared with EPDM/MMT-10, EPDM/DMMT-10 showed excellent filler dispersion and strong interfacial interactions. As a result, the mechanical performance of the EPDM/DMMT-10 was greatly enhanced. The highest tensile strength of the composites reached 6.5 MPa with 10 phr DMMT, almost 200% higher than that of pure EPDM. Moreover, the elongation at break of EPDM/DMMT-10 reached 720%, much higher than that in the literature. This study demonstrates the potential of MMT modification and the gel compounding method to prepare high-performance nanocomposites. These methods can improve the dispersion of inorganic fillers in the non-polar rubber matrix and enhance the interfacial strength of fillers and non-polar rubber. 

## Figures and Tables

**Table 1:** Formulations of EPDM composites.

Ingredient	Pure EPDM	EPDM/MMT-10	EPDM/DMMT-10
EPDM	100	100	100
VTMS	0	0	2
MMT	0	10	0
DMMT	0	0	10
ZnO	8	8	8
MgO	4	4	4
TAIC	2	2	2
DCP	4.5	4.5	4.5
4010NA	2	2	2

**Table 2:** Comparison of mechanical properties of various filler-filled EPDM composites.

Filler Type	Filler Loading (phr)	Tensile Strength (MPa)	Elongation at Break (%)	References
Carbon black	100	7.0	410	[42]
Silica	25	1.8	290	[42]
Aluminium hydroxide	50	7.2	315	[43]
Dendrimer-modified MMT	10	4.3	135	[44]
Octadecyl ammonium ion-modified MMT	10	4.2	320	[45]
DMMT	10	6.5	720	This work

## Data Availability

The original contributions presented in the study are included in the article, further inquiries can be directed to the corresponding authors.
